# A two-year mirror-image study of the effect of treatment with paliperidone and aripiprazole long-acting injections on need for inpatient care and home treatment intervention

**DOI:** 10.1192/bjo.2021.745

**Published:** 2021-06-18

**Authors:** Shay-Anne Pantall, Joseph Pilsbury, Le Gan, Lisa Brownell

**Affiliations:** 1 Birmingham and Solihull Mental Health NHS Foundation Trust; 2 University of Birmingham

## Abstract

**Aims:**

To evaluate the effect of the use of aripiprazole and paliperidone long acting injections on healthcare resource use

**Background:**

Long acting injections of second-generation antipsychotics such as paliperidone and aripiprazole have become more commonly prescribed over the past decade. They have much higher acquisition costs when compared to first generation depot antipsychotics. It is therefore essential to demonstrate their tolerability and cost-effectiveness.

**Method:**

We undertook an observational, retrospective two-year mirror study for all patients who started treatment with paliperidone long acting injection between January and June 2016 (n = 47) or aripiprazole long acting injection between April 2014 and July 2017 (n = 93). Clinical notes were examined to determine the number of admissions, inpatient days, home treatment episodes and number of home treatment days, in the 12 months preceding and following the commencement of the long acting injection.

**Result:**

70% remained on paliperidone and 62% remained on aripiprazole at the end of the one-year period.

There was a significant reduction in occupied bed days in those treated with paliperidone from 78.2 days in the year before this treatment was started to 25.4 days in the year after (p = 0.002). There was a significant reduction in occupied bed days in those treated with aripiprazole from 66.51 days to 32.7 days (p = 0.0006).

There was no significant reduction in days spent under the care of home treatment teams for individuals treated with either of these medicines.

**Conclusion:**

Treatment with either paliperidone or aripiprazole long-acting injection was associated with a reduction in admissions and occupied bed days of a magnitude that delivered an overall cost-saving despite the high drug acquisition costs. It remains to be determined how these reductions compare with other second-generation long-acting injections and first-generation depot antipsychotics.

